# Psychological, informational, logistical, and social determinants of regular blood donation in Malaysia

**DOI:** 10.3389/fpubh.2026.1881667

**Published:** 2026-09-08

**Authors:** Rabiatul Adawiyah Miswan, Sook Zhen Yong, Lois Xin Ning Low, Christina Lai Ling Lee, Li Ping Wong, Yulan Lin, Chenyun Zhang

**Affiliations:** 1 Centre for Population Health (CePH), Department of Social and Preventive Medicine, Faculty of Medicine, Universiti Malaya, Kuala Lumpur, Malaysia; 2 Department of Transfusion Medicine, Universiti Malaya Medical Centre, Kuala Lumpur, Malaysia; 3 Faculty of Medicine, Universiti Malaya, Kuala Lumpur, Malaysia; 4 Department of Medicine, Faculty of Medicine, Universiti Malaya, Kuala Lumpur, Malaysia; 5 Department of Epidemiology and Health Statistics, School of Public Health, Fujian Medical University, Fuzhou, Fujian, China; 6 Fujian Key Laboratory of Environmental Factors and Cancer, Fujian Medical University, Fuzhou, Fujian, China; 7 Department of Medicine, College of Medicine, Korea University, Seoul, Republic of Korea; 8 School of Health Management, Fujian Medical University, Fuzhou, Fujian, China

**Keywords:** barriers, blood donation, cross-sectional study, donor behavior, facilitators

## Abstract

**Background:**

Understanding donor profiles and the barriers and facilitators influencing blood donation is crucial for strengthening voluntary blood donation programs. This study aimed to examine the socio-demographic profile of blood donors and to identify the key barriers and facilitators influencing blood donation practices framed within psychological, informational, logistical, and social dimensions.

**Methods:**

A cross-sectional study was conducted among donors at the University Malaya Medical Centre (UMMC) using a structured questionnaire to assess sociodemographic characteristics and perceived barriers and facilitators of blood donation. Multivariable logistic regression identified independent predictors of donation behavior.

**Results:**

A total of 262 participants completed the study, of whom 73.0% were regular donors and 27.0% were first-time or non-regular donors. Multivariable analysis revealed several significant predictors of regular blood donation. Among demographic factors, married individuals had higher odds of donating regularly (aOR = 2.07, 95% CI: 1.02–4.20, *p* = 0.043). In contrast, participants with higher educational attainment were less likely to be regular donors, including those with a diploma (aOR = 0.13, *p* < 0.001) and those with a degree or above (aOR = 0.19, *p* = 0.002). High altruism and motivation were the strongest facilitators (aOR = 2.79, 95% CI: 1.39–5.61, *p* = 0.004). Conversely, limited knowledge of donation procedures (aOR = 0.31, 95% CI: 0.15–0.64, *p* = 0.001) and prior detrimental experiences (aOR = 0.21, 95% CI: 0.09–0.49, *p* < 0.001) significantly reduced the likelihood of regular donation.

**Conclusion:**

These findings highlight the strong influence of altruism, social factors, and donor experience on promote regular blood donation. Interventions should also specifically target single and highly educated individuals.

## Background

Blood donation is a cornerstone of modern healthcare, supporting life-saving interventions in trauma, surgery, oncology, obstetric emergencies, and chronic blood disorders, with millions of patients worldwide depending on transfusions each year to survive or improve their quality of life (1, 2). In Malaysia, the Ministry of Health estimates that a minimum of 2,000 blood donations are required each day to meet clinical needs; however, current donation rates remain below this target. This highlights ongoing challenges in achieving equitable access to blood donation opportunities. Although national blood collection has generally increased over the past decades, demographic transitions, growing healthcare demands, and an aging population continue to strain the overall blood supply system (3, 4).

Blood donation represents sustained prosocial health behavior shaped not only by awareness and accessibility, but also by psychological, informational, logistical, and social influences. From a behavioral medicine perspective, regular donation requires long-term motivation, positive prior experiences, trust in donation safety, and supportive social environments. Understanding these multidimensional determinants is essential for developing effective strategies to recruit and retain voluntary blood donors, particularly in middle income countries where maintaining a stable blood supply remains an ongoing public health challenge. Despite ongoing blood donation campaigns and increasing healthcare demands, maintaining a stable pool of regular voluntary blood donors remains a persistent challenge in Malaysia.

Much of the existing literature focuses on general donor awareness or donation intention, with limited understanding of the behavioral and psychosocial factors that sustain regular donation practices. In particular, there is insufficient evidence distinguishing regular from non-regular donors within hospital-based settings, where a substantial proportion of blood collection occurs. This gap limits the development of targeted strategies to improve long-term donor retention and ensure a sustainable blood supply. Previous studies underscore the multifaceted interplay of psychological, informational, logistical, and social factors that shape blood donation behavior, serving either as barriers or facilitators, and highlight the importance of developing context-specific strategies to enhance donor recruitment and long-term retention. Psychological barriers such as fear of needles, anxiety, and perceived health risks remain prevalent across different settings (5, 6). Informational barriers, including inadequate knowledge of eligibility criteria and donation procedures, also hinder participation (4). Logistical constraints, such as time limitations, distance to donation centers, and limited access to mobile drives further discourage potential donors (7). In contrast, altruism and personal motivation consistently emerge as the strongest facilitators, often cited as the primary reasons for voluntary donation (8, 9). Social norms, peer influence, and cultural or religious beliefs can also shape donation behavior, though their influence varies by context and population group (7, 10). While small incentives such as tokens or certificates may offer temporary motivation, they are less effective in fostering sustained donation practices (4). While this literature has advanced understanding of donor behavior, several important gaps remain. In Malaysia, most studies treat blood donors as a homogeneous group, without distinguishing between regular and non-regular donors (11). This represents a significant oversight, as evidence consistently shows that regular donors contribute disproportionately to blood supply stability, often donating multiple times per year and forming the backbone of national blood programs (3). By failing to differentiate donor types, this may underestimate the unique barriers faced by first-time or irregular donors and overlook the specific facilitators that sustain long-term commitment. In Malaysia, while public awareness campaigns have been widely implemented, their effectiveness remains limited among younger populations who are underrepresented in donor registries (12, 13). Few studies have examined the interplay between sociodemographic characteristics and the mix of psychological, informational, logistical, and social influences in shaping donation patterns within hospital-based populations, where much of Malaysia’s blood collection occurs (4, 14).

Despite the growing body of research on blood donation behavior, several key gaps persist. In Malaysia, most studies treat blood donors as a homogeneous group without distinguishing between regular and non-regular donors, limiting understanding of the unique motivations and barriers faced by each subgroup (3, 15). Regular donors contribute disproportionately to the national blood supply, yet the psychosocial and demographic determinants sustaining their continued participation remain underexplored. In addition, few studies have examined how sociodemographic characteristics interact with psychological, informational, logistical, and social factors to influence regular blood donation in hospital-based settings, where a substantial proportion of Malaysia’s blood collection occurs. Addressing these gaps is essential for developing targeted interventions to improve donor recruitment and long-term retention.

The present study aimed to examine the sociodemographic characteristics, barriers, and facilitators influencing blood donation practices within the framework of psychological, informational, logistical, and social factors. Specifically, it sought to: (1) describe the characteristics of blood donors; (2) identify the key barriers and facilitators affecting donation behavior; and (3) determine the predictors of regular blood donation across the psychological, informational, logistical, and social domains.

## Methods

### Study design and setting

A cross-sectional study was conducted at the Transfusion Medicine Department, University Malaya Medical Centre (UMMC), the largest tertiary teaching hospital in Malaysia. As a major tertiary referral center, UMMC represents an important hospital based blood donation setting where regular donor retention is essential for maintaining a stable blood supply. Eligible participants were Malaysian citizens aged ≥18 years who presented to the UMMC Blood Donation Centre during the study period. Non-Malaysians and those deferred on medical grounds were excluded.

### Sample size and sampling

Universal sampling was used to minimize selection bias by inviting all eligible donors attending the center during the study period to participate. The minimum sample size was calculated using OpenEpi (version 3.01), based on reported prevalence of regular donation ranging from 23.4 to 78.6% (16–18). With a 95% confidence level, 5% precision, and 20% allowance for non-response, the final target sample size was 343 participants.

### Study variables

The primary outcome was donor type. Regular donors were defined as individuals who had donated blood at least twice within the preceding 24 months, in accordance with the Malaysian Ministry of Health guideline (19). Non-regular donors were defined as those who did not meet this criterion, including both first-time donors and repeat donors with fewer than two donations within the previous 24 months (19). Independent variables included perceived barriers (e.g., limited knowledge, emotional barriers, time constraints, perceived risk, detrimental experience, accessibility) and facilitators (e.g., cultural/religious beliefs, social norms, altruism, incentives) assessed on a 5-point Likert scale. Sociodemographic characteristics (age, gender, race, religion, marital status, education, employment, income) and donation history were also collected. Age, gender, and education were included *a priori* as confounders.

### Data collection

Data were collected using a structured, self-administered questionnaire (Appendix 1) via mobile phone in the post-donation rest area. The questionnaire included multidimensional constructs covering behavioral, psychosocial, informational, and contextual factors associated with blood donation behavior. The questionnaire was built in REDCap, a secure HIPAA-compliant web platform. To minimize missing data, face-to-face administration was conducted when necessary. The questionnaire was adapted from validated tools (4, 20, 21) and modified for cultural relevance. Content validity was established through expert review by one public health specialist and three transfusion medicine specialists, yielding an Item-Level Content Validity Index (I-CVI) ranging from 0.67 to 1.00 and a Scale-Level Content Validity Index/Average (S-CVI/Ave) of 0.98. A pilot study involving 20 blood donors confirmed the clarity and feasibility of the questionnaire, and acceptable internal consistency was demonstrated (Cronbach’s *α* ≥ 0.70). Test–retest reliability using the intraclass correlation coefficient (ICC) was planned but not conducted due to time constraints and is noted as a study limitation.

### Data analysis

Data were analyzed in JASP 0.19.3.0. Descriptives summarized characteristics. Pearson’s χ^2^/Fisher’s exact test categorical associations. Variables with *p* < 0.25 in univariable screening entered multivariable logistic regression; results reported as aOR with 95% CI. Model fit was assessed using the Hosmer–Lemeshow goodness-of-fit test, and multicollinearity was evaluated using variance inflation factors (VIF).

## Results

A total of 262 blood donors participated in the study, giving a response rate of 76.4% (262/343). Of these, 191 (73.0%) were regular while 71 (27.0%) were non-regular donors (including first-time and infrequent repeat donors). The majority were male (62.2%), aged 30–49 years (62.0%), married (60.7%), and employed (76.7%). Most had attained a diploma or higher education (77.5%).

### Univariable analysis of socio-demographic and psychosocial factors

Table 1 showed that regular donation was significantly associated with age, marital status, education, and employment status. Older individuals were more likely to donate regularly (≥50 years: 83.9%) compared to younger donors (18–29 years: 60.9%). Married participants also had higher regularity (79.2%). Those with lower educational attainment donated more consistently (secondary or below: 89.8%). Among employment groups, self-employed donors showed the highest regular donation (92.9%). No significant associations were found for gender, race, religion, or household income.

**Table 1:** Comparison of donor characteristics, barriers, and facilitators between regular and non-regular donors.

Variable	Frequency (%)	Univariable analysis		*p*-value^a^
		Regular (*n* = 191)	Non-regular/First-time (*n* = 71)	
Socio-demographic characteristics				
*Age*				
18–29	69 (26)	42 (60.9)	27 (39.1)	0.021
30–49	162 (62)	123 (76)	39 (24)	
≥50	31 (12)	26 (83.9)	5 (16.1)	
*Gender*				
Male	163 (62.2)	125 (76.7)	38 (23.3)	0.077
Female	99 (37.8)	66 (66.7)	33 (33.3)	
*Race*				
Malay	172 (65.6)	120 (69.8)	52 (30.2)	0.119
Chinese	67 (25.6)	51 (76)	16 (24)	
Indian	18 (6.9)	17 (94.4)	1 (5.6)	
Others	5 (1.9)	3 (60)	2 (40)	
*Religion*				
Islam	174 (66.4)	122 (70)	52 (30)	0.307
Buddha	53 (20.2)	41 (77.4)	12 (22.6)	
Hindu	15 (5.7)	14 (93.3)	1 (6.7)	
Christian	15 (5.7)	10 (66.7)	5 (33.3)	
Others	5 (1.9)	4 (80)	1 (20)	
*Marital status*				
Currently not married	103 (39.3)	65 (63.1)	38 (36.9)	0.004
Married	159 (60.7)	126 (79.2)	33 (20.8)	
*Level of education*				
Secondary or below	59 (22.5)	53 (89.8)	6 (10.2)	0.003
Diploma/Certificate	91 (34.7)	60 (65.9)	31 (34.1)	
Degree and above	112 (42.8)	78 (69.6)	34 (30.4)	
*Working status*				
Unemployed/Retired/Student	33 (12.6)	21 (63.6)	12 (36.4)	0.027
Self-employed	28 (10.7)	26 (92.9)	2 (7.1)	
Employed	201 (76.7)	144 (71.6)	57 (28.4)	
*Household income*				
Low	128 (48.8)	92 (71.9)	36 (28.1)	0.721
Middle	89 (34)	64 (71.9)	25 (28.1)	
High	45 (17.2)	35 (77.8)	10 (22.2)	
Barriers				
*Limited knowledge*				
No	136 (52)	115 (84.6)	21 (15.6)	<0.001
Yes	126 (48)	76 (60.3)	50 (39.7)	
*Emotional barrier*				
No	208 (79.4)	161 (77.4)	47 (22.6)	0.001
Yes	54 (20.6)	30 (55.6)	24 (44.4)	
*Time constraints*				
No	147 (56.1)	122 (83)	25 (17)	<0.001
Yes	115 (43.9)	69 (60)	46 (40)	
*Perceived risk*				
No	182 (69.5)	139 (76.3)	43 (23.6)	0.056
Yes	80 (30.5)	52 (65)	28 (35)	
*Detrimental experience*				
No	186 (71)	152 (81.7)	34 (18.3)	<0.001
Yes	76 (29)	39 (51.3)	37 (48.7)	
*Accessibility*				
No	169 (64.5)	135 (80)	34 (20)	<0.001
Yes	93 (35.5)	56 (60)	37 (40)	
Facilitators				
*Cultural and religious beliefs*				
Low	150 (57.3)	108 (72)	42 (28)	0.704
High	112 (42.7)	83 (74.1)	29 (25.9)	
*Social norms and peer influence*				
Low	160 (61.1)	111 (69.4)	49 (30.6)	0.108
High	102 (38.9)	80 (78.4)	22 (21.6)	
*Altruism and motivation*				
Low	144 (55)	91 (63.2)	53 (36.8)	<0.001
High	118 (45)	100 (84.7)	18 (15.3)	
*Incentives*				
Low	156 (60)	107 (68.6)	49 (31.4)	0.057
High	106 (40)	84 (79.2)	22 (20.8)	

a Pearson’s chi-square was performed. Fisher’s exact test when expected cell counts were <5.

Significant associations were observed for limited knowledge (*p* < 0.001), emotional barriers (*p* = 0.001), time constraints (*p* < 0.001), detrimental experiences (*p* < 0.001), and accessibility (*p* < 0.001). Donors with limited knowledge were less likely to be regular (60.3%) compared to those without such barriers. Similarly, 44.4% of those reporting emotional barriers and 48.7% with detrimental experiences were non-regular donors. High altruism and motivation were significantly associated with regular donation (84.7%; p < 0.001).

### Perceived barriers to blood donation

**Figure 1:**
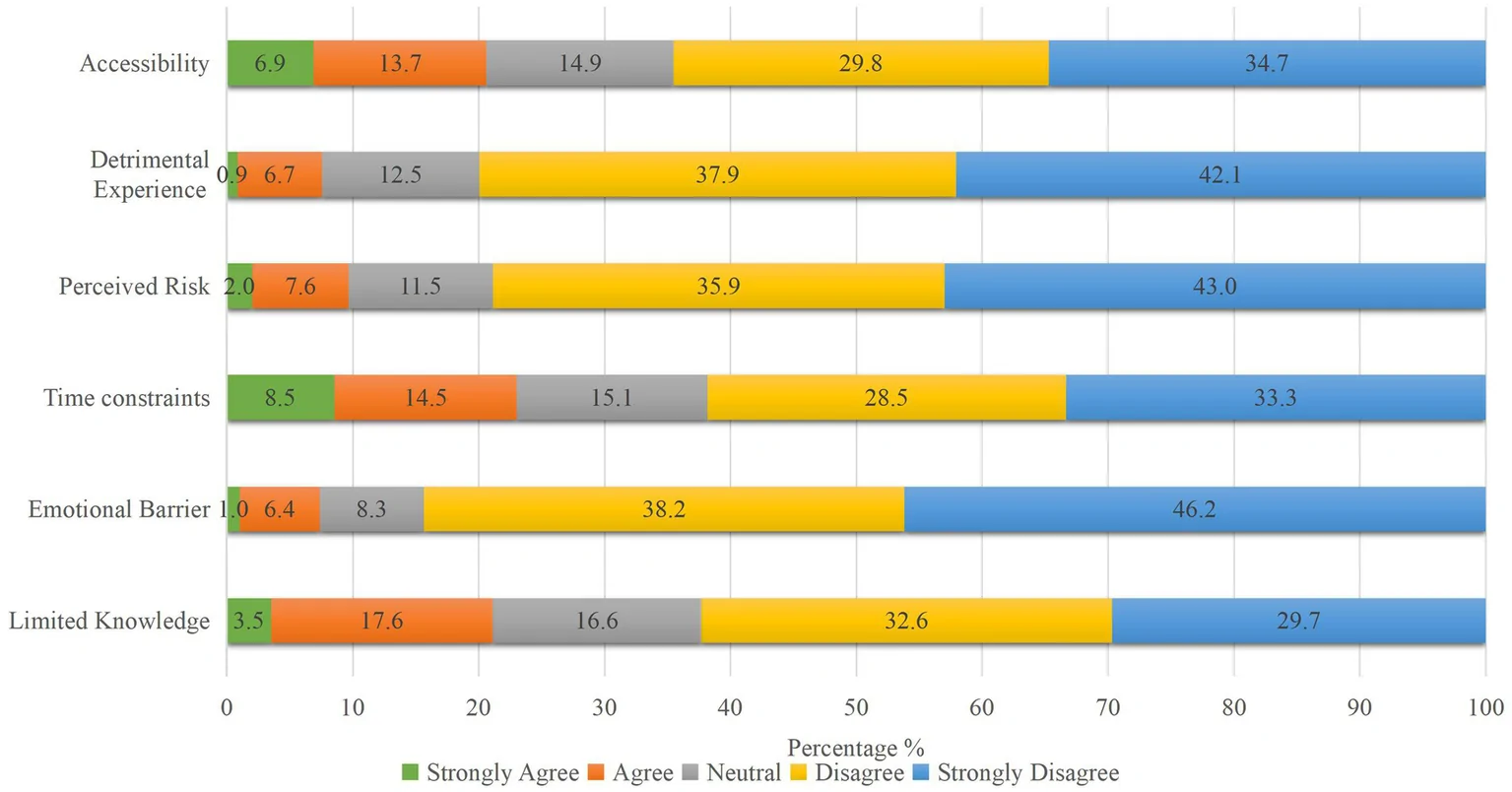
Distribution of response to barriers of blood donation.

Figure 1 illustrates the findings on the most frequently reported barriers to blood donation. Overall, emotional concerns such as fear of needles or anxiety were rarely reported (15.7%), with the vast majority dismissing them, suggesting that psychological resistance is not a major obstacle in this sample. In contrast, external or situational barriers were more prominent. Time constraints and accessibility issues were each acknowledged by over one-fifth of respondents, and when combined with those who expressed uncertainty, nearly one-third of participants viewed these as potential hindrances (38.1%). Knowledge-related barriers also emerged, with approximately one-quarter (37.7%) either admitting gaps or expressing uncertainty about the donation process, underscoring the need for continuous educational reinforcement. Perceived health risks were largely rejected (78.9%); however, a notable minority (around one-fifth) either feared potential harm or lacked confidence in donation safety, indicating persistent misconceptions. Finally, although detrimental prior experiences were uncommon (20.1%), their influence on a small subset of donors highlights the importance of ensuring safe and positive donation encounters to promote repeat donations (Table 2).

**Table 2:** Facilitators associated with regular blood donation.

Variable	Univariable analysis	*p*-value	Multivariable analysis (final model M₇)	*p*-value
	Crude OR (95% CI)		Regular vs. Non-regular/First-time blood donor aOR (95% CI)	
Socio-demographic characteristics				
*Age*				
18–29	Reference			
30–49	2.03 (1.11–3.71)	0.022		
≥50	3.34 (1.14–9.77)	0.027		
*Gender*				
Male	Reference			
Female	0.61 (0.35–1.06)	0.078		
*Race*				
Malay	Reference			
Chinese	1.38 (0.72–2.64)	0.329		
Indian	7.34 (0.96–56.82)	0.05		
Others	0.65 (0.11–4.0)	0.642		
*Religion*				
Islam	Reference			
Buddha	1.46 (0.71–2.99)	0.307		
Hindu	5.97 (0.77–46.56)	0.088		
Christian	0.85 (0.28–2.62)	0.78		
Others	1.705 (0.19–15.62)	0.637		
*Marital status*				
Currently not married	Reference		Reference	
Married	2.23 (1.28–3.89)	0.005	2.07 (1.02–4.20)	0.043
*Level of education*				
Secondary or below	Reference		Reference	
Diploma/Certificate	0.22 (0.09–0.57)	0.002	0.13 (0.05–0.39)	<0.001
Degree and above	0.26 (0.10–0.66)	0.005	0.19 (0.07–0.54)	<0.01
*Working status*				
Unemployed/Retired/Student	Reference		Reference	
Self-employed	7.43 (1.49–36.93)	0.014	3.92 (0.64–24.14)	0.14
Employed	1.44 (0.67–3.13)	0.352	0.80 (0.30–2.15)	0.664
*Household income*				
Low	Reference			
Middle	1.00 (0.55–1.83)	0.995		
High	1.37 (0.61–3.05)	0.442		
Barriers				
*Limited knowledge*				
No	Reference		Reference	
Yes	0.28 (0.15–0.5)	<0.001	0.31 (0.15–0.64)	<0.01
*Emotional barrier*				
No	Reference			
Yes	0.37 (0.2–0.68)	0.002		
*Time constraints*				
No	Reference			
Yes	0.38 (0.22–0.67)	<0.001		
*Perceived risk*				
No	Reference		Reference	
Yes	0.58 (0.32–1.02)	0.058	2.19 (0.92–5.21)	0.075
*Detrimental experience*				
No	4.24 (2.37–7.60)	<0.001	Reference	
Yes	Reference		0.21 (0.09–0.49)	<0.001
*Accessibility*				
No	Reference			
Yes	0.38 (0.22–0.67)	<0.001		
Facilitators				
*Cultural and religious beliefs*				
Low	Reference			
High	1.11 (0.64–1.94)	0.704		
*Social norms and peer influence*				
Low	Reference			
High	1.6 (0.90–2.87)	0.109		
*Altruism and motivation*				
Low	Reference		Reference	
High	3.24 (1.77–5.92)	<0.001	2.79 (1.39–5.61)	0.004
*Incentives*				
Low	Reference			
High	1.75 (0.98–3.12)	0.058		

Multivariable logistic regression results are presented for the final model (M₇) derived using stepwise selection.

### Perceived facilitators of blood donation

**Figure 2:**
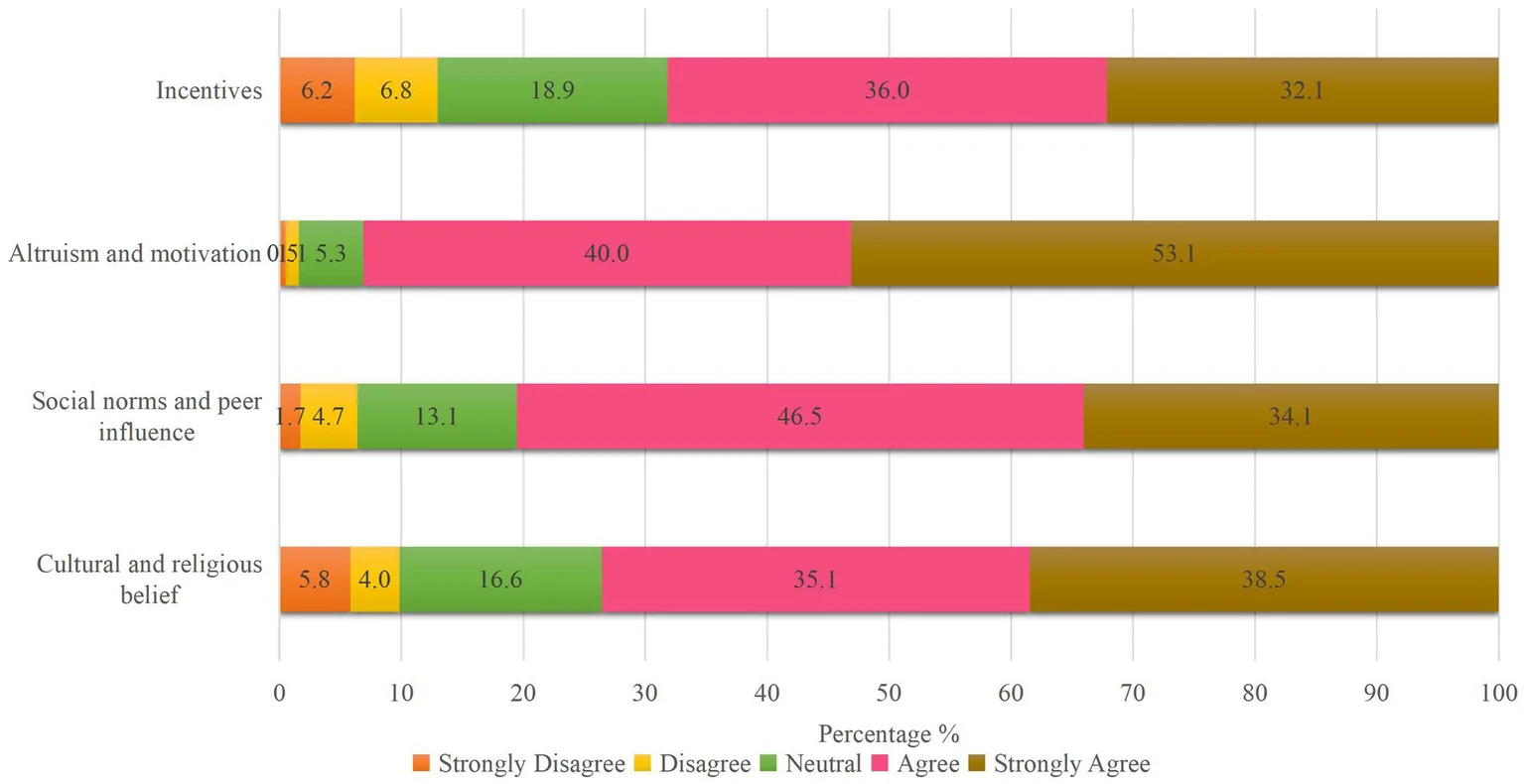
Perceived facilitators of blood donation among participants.

Findings on the most frequently reported facilitators of blood donation are shown in Figure 2. Altruism and motivation emerged as the most common factors, with the vast majority of respondents (93.1%) affirming that helping others was their primary driver. In contrast, social norms and peer influence were less consistently endorsed, with fewer than two-thirds agreeing, suggesting that external social pressures may play a more modest role in shaping donor behavior. Cultural and religious beliefs showed a mixed response, with just over half perceiving them as facilitators (73.6%), while a sizeable proportion remained neutral or disagreed, indicating variability in their perceived importance across the sample. Incentives such as tokens or small rewards were the least persuasive, with opinions almost evenly divided between agreement and disagreement, highlighting that extrinsic rewards may not strongly motivate donation compared with intrinsic values like altruism.

### Barriers and facilitators predicting regular blood donation

In the multivariable analysis, the strongest positive predictor was high altruism (aOR = 2.79), indicating nearly three times higher odds of being a regular donor, followed by marital status (aOR = 2.07; 95% CI: 1.02–4.20; *p* < 0.05) and perceived safety (aOR 2.19; 95% CI: 0.92–5.21; *p* = 0.075), though the latter only approached significance. On the other hand, the strongest negative predictor was detrimental experiences (aOR 0.21; 95% CI: 0.09–0.49; *p* < 0.001), which reduced the odds of regular donation by almost 80%. Educational attainment also showed a strong inverse association, with diploma holders (aOR 0.13; 95% CI: 0.05–0.391; *p* < 0.001) and degree holders (aOR = 0.19; 95% CI: 0.07–0.54; *p* < 0.1) substantially less likely to donate compared with those with lower education. Limited knowledge (aOR 0.31; 95% CI: 0.15–0.64; *p* < 0.01) was another important barrier, reducing the odds of regular donation by nearly 70%. By demographic, educational attainment also showed a strong inverse association, with diploma holders (aOR 0.13; 95% CI: 0.05–0.39; *p* < 0.001) and degree holders (aOR 0.19; 95% CI: 0.07–0.54; *p* < 0.01) substantially less likely to donate compared with those with lower education. Married individuals had over twice the odds (OR 2.23; 95% CI: 1.28–3.89; *p* = 0.05).

The stepwise multiple logistic regression showed progressive improvement in model fit, with the final model (M₇) demonstrating good explanatory power (Nagelkerke R^2^ = 0.358, Cox and Snell R^2^ = 0.246, Tjur R^2^ = 0.282), explaining about one-third of the variance in regular blood donation. Multicollinearity was not a concern, as VIF values ranged from 1.03 to 1.72 and tolerance values exceeded 0.58, all within acceptable thresholds.

## Discussion

This study provides important insights into the key factors that influence donor behavior and offers guidance for strategies to improve donor recruitment and retention. The findings emphasize that donor behavior is shaped by an intricate mix of psychological, informational, logistical, and social influences. Importantly, the findings reinforce the view of blood donation as a sustained prosocial health behavior that is strongly influenced by behavioral and social contexts beyond simple awareness or access to services.

The majority of participants in this study were regular donors, while slightly more than one-quarter were first-time or non-regular donors. This pattern underscores the crucial role of regular donors in maintaining a safe, stable, and sufficient blood supply. Regular donors not only contribute to the reliability and efficiency of blood services but are also associated with a lower risk of transfusion-transmissible infections, thereby improving the overall safety of the blood supply (22). The relatively high proportion of regular donors observed in this study is encouraging and may reflect the effectiveness of existing donor retention efforts within the hospital’s blood donation program. However, the notable presence of first-time and non-regular donors indicates opportunities for further improvement. These groups represent a critical target for interventions designed to convert them into repeat donors. Focused educational campaigns, personalized donor engagement strategies, and improved donor experience, such as streamlined registration, reduced waiting time, follow-up reminders, could enhance their motivation and long-term commitment. For instance, one study found that reminder communications (telephone or SMS) significantly supported donor re-activation and retention (23). Similarly, evidence from China indicates that structured post-donation follow-up system improved repeat donation rates, suggesting that systematic engagement plays a critical role in donor retention (24).

When viewed within an international context, the prevalence of regular donors observed in this study mirrors trends reported in several high-income countries. In the United States, approximately 74% of donations in 2021 were from repeat donors, reflecting a strong culture of voluntary and repeat donation (25). Similarly, Western European countries have achieved even higher proportions, around 84% overall and up to 95% in Netherlands, largely due to long-established donor management systems and sustained public trust in blood services (2). By contrast, many LMICs continue to depend heavily on first-time and replacement donors, highlighting persistent structural and sociocultural barriers to donor retention. For example, a study in rural India found that 73% of donors were first-time participants (26), while research from Central Africa reported that over 70% of blood donations originated from first-time donors (27). These findings align with broader global evidence demonstrating that LMICs face challenges in establishing sustainable voluntary donor pools due to limited infrastructure, inconsistent follow-up, and weaker social marketing systems (28). This suggests that donor retention represents not only a transfusion service challenge, but also a broader behavioral and public health issue, particularly in LMIC settings.

By demographic characteristics, the majority of regular donors in this study were male, aged 30–49 years, married, and employed. The demographic profile of our participants was also generally consistent with recent Malaysian blood donor registry statistics, which show that blood donors are predominantly male (approximately 60–63%) with a mean age of around 31 years. Our study similarly comprised predominantly male donors (62.2%), and the age profile was broadly comparable with the national donor population (29). These similarities suggest that our sample is broadly representative of the demographic characteristics of Malaysian blood donors. Nevertheless, because participants were recruited from a single tertiary hospital, caution is still warranted when generalizing the findings to all blood donors in Malaysia. In this study, older significantly associated with regular blood donation, with donors aged 50 years and above being more than three times as likely to donate regularly compared with those in the 18–29 age group. This pattern aligns with global evidence showing that blood donation is more prevalent among individuals who are older, socially stable, and economically active (30, 31). Comparable patterns have been observed in China. Data from Zhejiang Province (2006–2015) show that donors were mainly young men aged 18–25 years at first, but by 2015 the dominant donor group had shifted to those aged 26–45 years (32), suggesting that younger adults face barriers such as busy lifestyles, stress, and misconceptions that limit their willingness to donate. In terms of gender, studies across diverse settings have consistently found higher participation in blood donation among men and middle-aged adults. This is largely attributed to gender differences in perceived eligibility, donation anxiety, and health-related restrictions that disproportionately affect women, such as pregnancy and menstruation. Women tend to experience greater anxiety about the donation process and have higher rates of adverse reactions, which negatively influence their likelihood of returning to donate. Additionally, health restrictions like low hematocrit and iron levels lead to more frequent deferrals of women from donating, whereas men are more often deferred for low blood pressure. These factors combined contribute to the observed gender gap in blood donation participation rates (33, 34). By age, this finding is consistent with evidence from Malaysia and other countries, where younger individuals often demonstrate greater hesitancy toward blood donation (31, 35). Several factors may explain this trend. Younger people commonly report needle fear, anxiety about side effects, and limited knowledge of donation procedures. In addition, misinformation, particularly through social media, can amplify these concerns. They may also have fewer opportunities or exposure to donation campaigns compared to older adults, who more often have established habits of civic participation. These findings highlight the need for targeted interventions to increase donation rates among younger populations, such as school- and university-based awareness programs, social media campaigns to counter misinformation, and initiatives that normalize donation as a routine, socially valued practice.

Two noteworthy findings emerged from this study. First, individuals with lower educational attainment were more likely to be regular blood donors. This pattern perhaps reflects stronger altruistic or community-oriented motivations among those with less formal education, consistent with previous reports that lower and middle educational groups tend to exhibit higher donation participation (36). Second, individuals with higher educational attainment were less likely to be regular blood donors. Although the underlying reasons cannot be determined from this cross-sectional study, several plausible explanations have been proposed in the literature. Individuals with higher educational attainment may face greater professional responsibilities, heavier workloads, and time constraints, limiting opportunities to donate despite positive attitudes toward blood donation. They may also exhibit different health-seeking behaviors or greater concern about potential health risks associated with blood donation (37, 38). In addition, work-related factors that were not measured in the present study, such as occupation type, workplace demands, commuting distance, and flexibility to attend donation sessions, may also contribute to the observed association. Educational differences in exposure and response to health communication may also influence participation, as lower educated individuals may be more responsive to community-based drives and peer recruitment (39). These findings highlight the importance of tailoring donor engagement strategies according to educational level. For individuals with higher education, initiatives should focus on workplace-based campaigns and more flexible donation opportunities, such as mobile donation units and extended operating hours, to accommodate work commitments. In contrast, for those with lower educational backgrounds, community outreach and peer-led programs may be more effective in sustaining participation and promoting long-term donor commitment.

Married individuals had twice the adjusted odds of being regular blood donors, underscoring the influence of social stability and responsibility on prosocial behavior. Marriage may strengthen a sense of social obligation and facilitate encouragement from spouses or family members, aligning with the “social embeddedness” theory of altruism, which emphasizes the role of interpersonal relationships in sustaining helping behaviors (40). These findings suggest that family- or couple-oriented recruitment strategies could be leveraged to enhance donor motivation and retention by reinforcing the positive social and emotional support associated with blood donation.

Findings on the most frequently reported barriers indicate that logistical and knowledge-related factors were the main obstacles to blood donation. Consistent with prior research, logistical barriers such as time constraints and limited accessibility remain major deterrents, especially among working adults balancing professional and personal commitments (41). Long travel times and fixed donation schedules, especially in urban areas, often discourage repeat donations in China (42). Wang (43) conducted a qualitative study in North China, which is representative of blood donation mobilization challenges in smaller cities and rural areas. Their findings highlighted the increased use of mobile blood donation drives to collect blood more efficiently, especially extending activities to rural areas by mobilizing farmers and organizing group donations at workplaces. This approach helped alleviate accessibility barriers due to limited blood collection sites in rural settings and improved blood supply during the COVID-19 pandemic. These findings emphasize the need for flexible donation systems, including mobile collection units, extended hours, and workplace-based programs to improve convenience. Two important findings related to knowledge and perceptions also emerged. First, knowledge gaps and misconceptions about blood donation persisted, particularly regarding donation procedures, which may undermine donor confidence (44). Similarly, a cross-sectional study among undergraduate students in the United Arab Emirates by Babker et al. (45) reported important knowledge gaps, misconceptions, and suboptimal blood donation practices, highlighting that educational deficits remain common among young adults. These findings reinforce the importance of targeted educational interventions to improve awareness, correct misconceptions, and encourage future blood donation. Secondly, barriers to regular donation are not solely structural, but also behavioral and cognitive, involving confidence, perceived safety, and familiarity with the donation process. Although psychological barriers such as fear and anxiety were less pronounced, their residual presence suggests that continued reassurance and transparent communication about safety and eligibility remain essential.

Findings on most frequently reported facilitators highlight that altruism remains the dominant motivator for blood donation. The strong endorsement of helping others as a primary reason for donation aligns with long-standing evidence that intrinsic motivations—such as empathy, moral duty, and a desire to contribute to the welfare of others—are the most consistent predictors of donor behavior across diverse populations (41, 46, 47). Altruism not only motivates initial donation but also buffers against deterrents such as fear, inconvenience, or prior negative experiences, highlighting its protective effect in maintaining long-term donor engagement (48). From a behavioral medicine perspective, these findings suggest that sustained donation behavior is driven more strongly by internalized prosocial values and positive reinforcement than by external incentives. In contrast, social norms and peer influence were less influential, suggesting that while social encouragement can initiate donation, sustaining regular participation depends more on internalized prosocial values than external pressure. Cultural and religious beliefs were perceived variably, reflecting contextual differences in how moral and faith-based values are linked to altruism and community responsibility. Finally, incentives such as tokens or small rewards appeared least effective, supporting previous evidence that extrinsic motivators have limited and short-term impact on donor retention compared with intrinsic satisfaction derived from altruistic acts (49, 50).

Results from the multivariable analysis highlight crucial predictors that can inform evidence-based interventions to enhance regular blood donation. The results highlight the strong influence of altruism, social factors, and donor experience on sustaining regular blood donation. High altruism emerged as the strongest positive predictor, aligning with extensive evidence that intrinsic motivations such as empathy, moral responsibility, and the desire to help others are the most consistent and enduring drivers of donation behavior. Perceived safety, though marginally significant, reflects the continuing importance of trust in blood collection systems and confidence in donation safety. Conversely, detrimental prior experiences represented the strongest negative predictor, indicating that even a single adverse event can substantially reduce willingness to donate again, as documented in multiple studies emphasizing the psychological impact of discomfort or fainting on donor return (51). Limited knowledge also significantly reduced donation likelihood, echoing evidence that poor awareness of eligibility, procedures, and safety undermines donor confidence (52). Similarly, Li et al. (50) found that among whole blood donors in China, fear of donation significantly reduced the intention to donate again, while higher self-efficacy increased it, suggesting that managing fear and building donor confidence are key to improving donor retention.

Taken together, the findings demonstrate that sustaining regular blood donation requires more than increasing donor recruitment alone. Long term donor retention depends on positive donation experiences, trust in donation safety, supportive social environments, and sustained altruistic motivation. These findings highlight the important contribution of behavioral medicine in understanding and improving voluntary blood donation practices.

The present findings also have important implications for blood donation promotion strategies. Communication campaigns should be tailored to population groups with lower participation, particularly highly educated and single individuals. For highly educated donors, convenience-focused approaches, such as workplace blood donation drives, extended operating hours, and appointment-based donation systems, may help overcome occupational and time-related barriers. Communication messages should also reinforce the safety of blood donation using clear, evidence-based information to address potential health concerns. For single individuals, campaigns that emphasize altruism, social responsibility, and peer engagement may help strengthen motivation to donate regularly. Such targeted approaches may be more effective than broad, one-size-fits-all campaigns in promoting long-term donor retention.

### Limitation

This study has several limitations. First, the cross sectional design limits causal interpretation of the observed associations. Second, as the study was conducted in a single hospital based setting among individuals who had already presented for blood donation, selection bias is possible. Participants who attend a donation center are likely to be more motivated, health-conscious, and positively inclined towards blood donation than those who do not present to donate. Consequently, the barriers identified in this study may underestimate those experienced by non-attenders or individuals unwilling to donate, and the findings should not be generalized to the broader population without caution. Self reported responses may also be influenced by recall or social desirability bias, particularly for altruistic motivations and attitudes toward donation. Furthermore, although we adjusted for several sociodemographic and behavioral factors, residual confounding from unmeasured psychosocial constructs, such as health literacy, trust in the healthcare system, and general health-seeking attitudes, cannot be excluded. These unmeasured factors may have influenced the observed associations, and therefore the reported effect sizes should be interpreted with appropriate caution. In addition, the test–retest reliability of the questionnaire was not assessed due to time constraints. Nevertheless, the study provides important behavioral insights into regular blood donation within a Malaysian teaching hospital context. Future multicenter and longitudinal studies involving both donors and non-donors are warranted to further examine donor retention behaviors across different populations and settings.

## Conclusion

This study highlights that regular blood donation is influenced by a complex interplay of demographic, psychological, informational, and social factors. Altruism and positive donor experiences emerged as important facilitators of regular donation, while limited knowledge and detrimental prior experiences were key barriers. The findings underscore the importance of strengthening donor education, fostering positive donation experiences, and promoting supportive social motivations to improve long term donor retention. Greater attention should also be directed toward engaging single and highly educated individuals, who were less likely to donate regularly, to support a more sustainable and diverse blood donor pool.

## Data Availability

The datasets presented in this study can be found in online repositories. The names of the repository/repositories and accession number(s) can be found in the article/Supplementary material.
