# Supplementary material for: Psychological, informational, logistical, and social determinants of regular blood donation in Malaysia
Source: Front Public Health. 2026 Sep 8;14:1881667. doi: 10.3389/fpubh.2026.1881667 (PMC13593761; doi:10.3389/fpubh.2026.1881667)
Supplement: Supplementary file 1 [file Supplementary_file_1.DOCX]

# Appendix 1: Questionnaire

**DONOR PROFILE, BARRIERS, AND FACILITATORS OF BLOOD DONATION PRACTICES**

## Section A: Sociodemographic Information

1. 1. Gender / Jantina:

☐ Male / Lelaki

☐ Female / Perempuan

2. 2. Age Group / Umur:

☐ 18–29 years

☐ 30–39 years

☐ 40–49 years

☐ 50 years and above

3. 3. Marital Status / Status Perkahwinan:

☐ Single / Bujang

☐ Married / Berkahwin

☐ Divorced / Bercerai

☐ Widowed / Balu/Duda

4. 4. Education Level / Tahap Pendidikan:

☐ Secondary or below / Menengah atau ke bawah

☐ Diploma/Certificate / Diploma/Sijil

☐ Degree and above / Ijazah dan ke atas

5. 5. Employment Status / Status Pekerjaan:

☐ Employed / Bekerja

☐ Self-employed / Bekerja sendiri

☐ Student / Pelajar

☐ Unemployed / Tidak bekerja

6. 6. Monthly Income / Pendapatan Bulanan:

☐ < RM3000

☐ RM3000–4999

☐ RM5000–9999

☐ ≥ RM10000

## Section B: Blood Donation History

7. 1. Have you donated blood before? / Pernahkah anda menderma darah sebelum ini?

☐ Yes / Ya

☐ No / Tidak

8. 2. How many times have you donated in the past 24 months? / Berapa kali anda telah menderma dalam tempoh 24 bulan lepas?

☐ None

☐ Once

☐ Twice

☐ Three or more times

9. 3. What motivates you to donate blood? / Apakah yang mendorong anda untuk menderma darah?

☐ Altruism / Altruisme

☐ Peer Influence / Pengaruh rakan

☐ Religious/Cultural Beliefs / Kepercayaan agama/budaya

☐ Incentives / Insentif

☐ Others (specify): ___________

## Section C: Perceived Barriers to Blood Donation

Please tick (✓) the level that best represents your agreement. / Sila tandakan (✓) tahap persetujuan anda.

1. Fear of needles or pain / Takut jarum atau kesakitan

☐ Strongly Disagree / Sangat Tidak Setuju	☐ Disagree / Tidak Setuju	☐ Neutral / Neutral	☐ Agree / Setuju	☐ Strongly Agree / Sangat Setuju

2. Lack of time / Kekangan masa

☐ Strongly Disagree / Sangat Tidak Setuju	☐ Disagree / Tidak Setuju	☐ Neutral / Neutral	☐ Agree / Setuju	☐ Strongly Agree / Sangat Setuju

3. Accessibility issues / Kesukaran akses ke pusat derma darah

☐ Strongly Disagree / Sangat Tidak Setuju	☐ Disagree / Tidak Setuju	☐ Neutral / Neutral	☐ Agree / Setuju	☐ Strongly Agree / Sangat Setuju

4. Limited knowledge about the donation process / Pengetahuan terhad tentang proses derma darah

☐ Strongly Disagree / Sangat Tidak Setuju	☐ Disagree / Tidak Setuju	☐ Neutral / Neutral	☐ Agree / Setuju	☐ Strongly Agree / Sangat Setuju

5. Perceived health risks / Risiko kesihatan yang dirasakan

☐ Strongly Disagree / Sangat Tidak Setuju	☐ Disagree / Tidak Setuju	☐ Neutral / Neutral	☐ Agree / Setuju	☐ Strongly Agree / Sangat Setuju

6. Previous negative donation experience / Pengalaman derma yang tidak baik

☐ Strongly Disagree / Sangat Tidak Setuju	☐ Disagree / Tidak Setuju	☐ Neutral / Neutral	☐ Agree / Setuju	☐ Strongly Agree / Sangat Setuju

## Section D: Perceived Facilitators of Blood Donation

1. Helping others / Membantu orang lain (Altruism)

☐ Strongly Disagree / Sangat Tidak Setuju	☐ Disagree / Tidak Setuju	☐ Neutral / Neutral	☐ Agree / Setuju	☐ Strongly Agree / Sangat Setuju

2. Peer encouragement / Galakan daripada rakan

☐ Strongly Disagree / Sangat Tidak Setuju	☐ Disagree / Tidak Setuju	☐ Neutral / Neutral	☐ Agree / Setuju	☐ Strongly Agree / Sangat Setuju

3. Religious or cultural beliefs / Kepercayaan agama atau budaya

☐ Strongly Disagree / Sangat Tidak Setuju	☐ Disagree / Tidak Setuju	☐ Neutral / Neutral	☐ Agree / Setuju	☐ Strongly Agree / Sangat Setuju

4. Trust in donation safety / Keyakinan terhadap keselamatan derma darah

☐ Strongly Disagree / Sangat Tidak Setuju	☐ Disagree / Tidak Setuju	☐ Neutral / Neutral	☐ Agree / Setuju	☐ Strongly Agree / Sangat Setuju

5. Receiving small tokens or rewards / Menerima cenderamata atau ganjaran kecil

☐ Strongly Disagree / Sangat Tidak Setuju	☐ Disagree / Tidak Setuju	☐ Neutral / Neutral	☐ Agree / Setuju	☐ Strongly Agree / Sangat Setuju
